# Affordable realtime respiratory monitoring system for radiation oncology

**DOI:** 10.1002/acm2.70250

**Published:** 2025-08-31

**Authors:** Mandeep Kaur, Hania Al‐Hallaq, Marian Axente

**Affiliations:** ^1^ Department of Radiation Oncology Emory University School of Medicine Atlanta Georgia USA

**Keywords:** motion management, optical imaging, respiratory motion monitoring

## Abstract

**Background and purpose:**

Managing respiratory motion is essential for accurate external beam radiotherapy of thoracic and abdominal tumors. Surface‐guided radiation therapy systems offer precise respiratory motion monitoring, albeit being often expensive and complex. Cost‐effective optical imaging technology could increase accessibility to accurate respiratory monitoring in radiotherapy.

**Materials and methods:**

A realtime respiratory monitoring system was developed using off‐the‐shelf depth camera and custom software. The system tracks a superficial surrogate in three dimensions using depth and optical data. Initial testing involved tracking rigid phantoms in static and dynamic scenarios. 1D sinusoidal motion of various amplitudes was used as ground truth to assess system performance at different distances from the camera. 3D‐motion was tracked concomitantly with the proposed system and a clinical surface imaging system. System accuracy against nominal motion was evaluated using Pearson correlation (r), tracking success rate (TSR), and mean absolute error (MAE). Bland–Altman analysis assessed agreement with the clinical system in the absence of ground truth.

**Results:**

The system accurately measured static targets, with depth errors of 0.03 ± 0.13 mm at 560 mm and 0.65 ± 0.61 mm at 1120 mm. For vertical 1D‐motion, TSR (≤1 mm) was 100% at 560 mm and 98.7% at 1120 mm; longitudinal TSR ranged from 92% to 50% as distance increased. Pearson correlation remained > 0.98, except at distances ≥980 mm and amplitudes ≤5 mm. Mean errors were consistently ≤1 mm, with minimal bias and narrow limits of agreement in Bland–Altman analysis compared to the clinical system.

**Conclusion:**

The proposed system demonstrates suitability for realtime respiratory motion monitoring in radiotherapy, while minimizing the implementation costs.

## 1 INTRODUCTION

Effective management of respiratory motion is crucial in external beam radiotherapy for thoracic and abdominal tumors, where breathing movements significantly impact tumor positioning and dosimetric accuracy.^1^, ^2^, ^3^ Inadequate monitoring can lead to unintended radiation exposure of both tumors and surrounding normal tissues.^4^, ^5^ Techniques such as forced shallow breathing with abdominal compression, breath‐hold, respiratory gating, and tracking are employed to minimize tumor motion, enhancing treatment accuracy and reducing unnecessary exposure to critical organs.^3^, ^6^, ^7^, ^8^, ^9^, ^10^, ^11^, ^12^, ^13^, ^14^, ^15^, ^16^, ^17^, ^18^, ^19^ However, these strategies can be compromised by factors like additional imaging dose, extended treatment durations, patient compliance issues, variability in respiratory motion, and technical implementation challenges.^2^, ^20^

Optical imaging using stereoscopic cameras and photogrammetry has emerged as a robust method for tracking respiratory motion, potentially overcoming existing challenges and enhancing treatment precision in radiotherapy.^21^, ^22^, ^23^, ^24^, ^25^, ^26^, ^27^, ^28^, ^29^, ^30^, ^31^, ^32^, ^33^ As a non‐invasive and non‐ionizing modality, it provides realtime monitoring of the chest and abdominal surfaces without additional radiation dose.^32^ Despite their benefits, high costs limit adoption, especially in resource‐constrained hospitals and clinics that still rely on conventional radiation therapy methods.^34^, ^35^ Smartphone based applications have been explored as a more accessible option,^36^, ^37^, ^38^ but these often require specialized hardware and are limited to 1D motion tracking.^38^

Previous approaches with depth sensors showed promising results but faced limitations under certain setup conditions: accuracy decreased with larger motion amplitudes and on curved surfaces impacting clinical effectiveness.^39^, ^40^, ^41^, ^42^, ^43^ Additionally, some approaches have lacked realtime data processing and relied on fixed regions of interest (ROIs), limiting their ability to adapt to changes in patient positioning or motion during treatment.^43^

A cost‐effective, real‐time respiratory monitoring system was developed using an off‐the‐shelf depth camera. This system leverages a stereoscopic camera providing simultaneous optical and depth data, similar to current optical imaging systems in radiotherapy. By leveraging computer vision techniques, the prototype dynamically tracks respiratory motion, adapting to changes in the target area for accurate monitoring. The proposed system aims to make respiratory motion management in radiotherapy more accessible while ensuring accuracy and easy implementation. Herein, we detail development details along with extensive testing and validation methodology and results of the system against ground truth and gold standard clinical systems.

## 2 MATERIALS AND METHODS

### 2.1 Depth sensing and MASK R‐CNN for system development

A realtime monitoring system was developed using the affordable Intel® RealSense™ D455 depth camera (Intel Corporation, Santa Clara, CA, USA) and a Mask R‐CNN algorithm^44^ for object detection and segmentation. A data pipeline was coded to utilize camera depth and optical inputs for realtime tracking of a triangle‐shaped sticker affixed on the tracked surfaces during live acquisition.

To train the Mask R‐CNN model to detect and segment triangle stickers against various backgrounds, we collected images featuring triangles of different colors, sizes, and orientations on backgrounds representing a range of skin tones. Image augmentation techniques such as random rotations, scaling, and flipping were applied to enhance model robustness. The trained model's detection accuracy was quantified using mean average precision (mAP).^45^ The final model achieved a mAP of 1.0 on the test dataset, indicating that the network reliably identifies the triangular marker.

The system initializes by configuring TensorFlow and the Mask R‐CNN framework for object detection and image analysis. The Intel® RealSense™ SDK 2.0 Python wrapper started the camera pipeline, facilitating communication with the hardware. Custom algorithms filter live depth and color data streamed at suitable resolution (848 × 480 pixels) and frame rate (30fps). After region of interest (ROI) selection via the graphical user interface (GUI) and application of filtering algorithms, the effective temporal sampling rate is reduced to approximately 10 Hz. Specifically, the acquisition firmware applies a five‐frame median filter to smooth single frame anomalies while maintaining data integrity. Contrast enhancement techniques like contrast limited adaptive histogram equalization (CLAHE)^46^ were applied to address ambient light variations in data acquisition scenarios.

After pre‐processing, a trained Mask R‐CNN algorithm is employed to detect triangular markers within the defined region of interest (ROI) in each frame of the color video. The centroids of these detected triangles are then calculated, and their position over time provides a real‐time series that captures the surrogate motion signal. Although the system uses a single triangle‐shaped sticker, which captures motion from a localized patch of the patient's surface, this sticker is deliberately placed on a relatively rigid region of the thoracic or upper abdominal area where in‐plane deformation during normal respiration is minimal. This placement ensures that the surrogate reliably reflects respiratory excursion amplitude, which is the primary target of motion tracking in this work. The 2D centroid coordinates are subsequently converted to 3D physical space using depth measurements, following the transformation described by Fielding et al.^43^

### 2.2 Depth measurement precision and drift of the camera system

**FIGURE 1:**
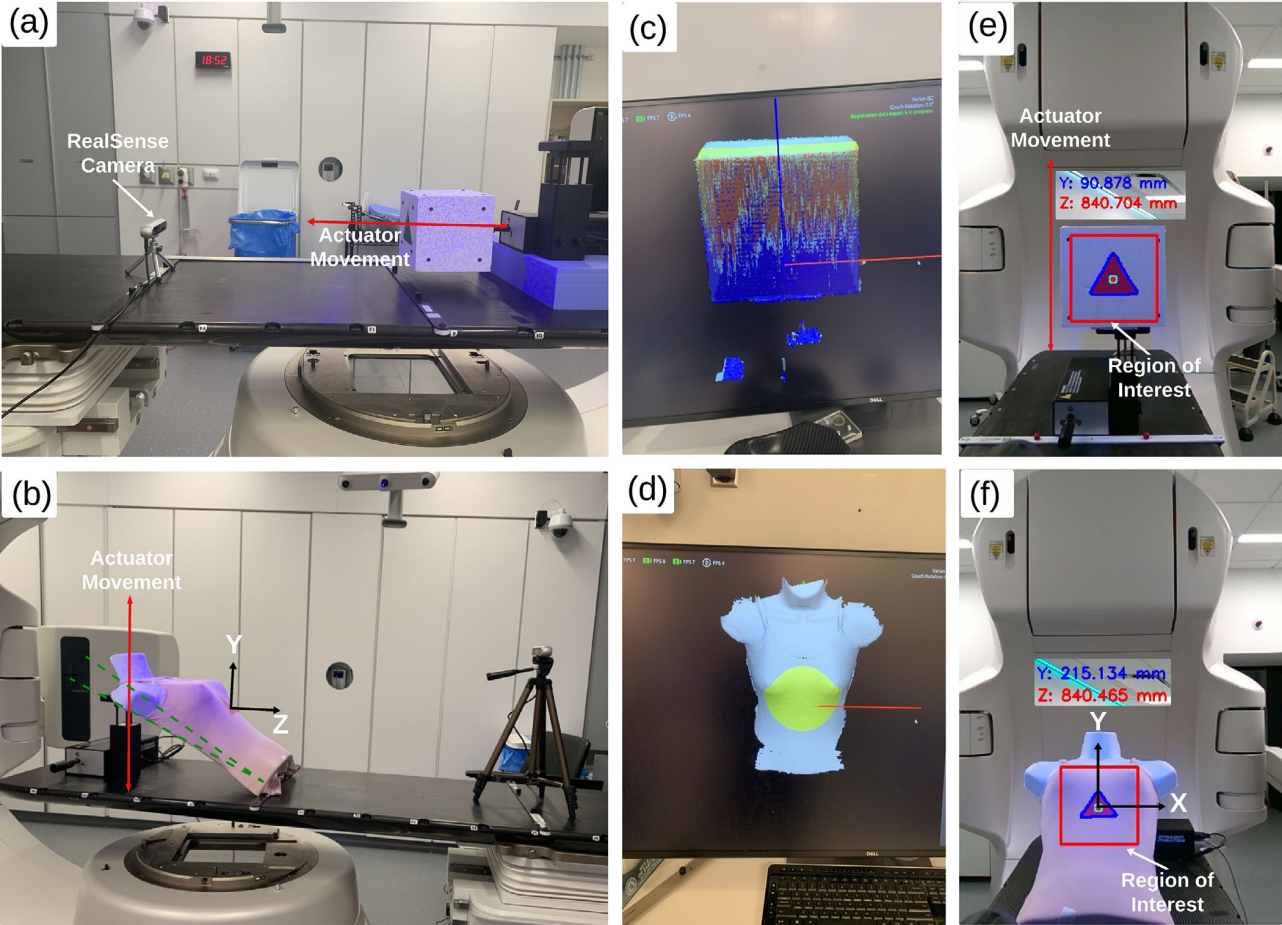
Camera system setup and motion monitoring (a) flat surface experiment for longitudinal motion; (b) mannequin torso with motion actuator (fixed at bottom motion pivot point); (c, d) Clinical system visualization (VARIAN IDENTIFY™) for flat surface and mannequin torso; (e) camera field‐of‐view with ROI tracking vertical motion of flat surface; (f) 3D tracking of moving mannequin torso within field‐of‐view.

Initial tests assessed the camera's depth sensing accuracy at varying distances from a flat surface with a triangle sticker affixed. The surface was aligned perpendicular to the camera's principal axis on the treatment couch of a TrueBeam medical linear accelerator (Varian Medical Systems, Inc., Palo Alto, CA, USA), using predefined indexes at distances from 560 to 1120 mm in 140 mm increments (Figure 1a). The algorithm executed for 1 min at each distance, capturing 600–700 frames per iteration to calculate the static object distance. To evaluate drift and system stability over time, a 130‐min test was conducted on a stationary surface at these predefined distances.

### 2.3 Motion tracking of flat surface and latency measurement

Dynamic tests employed the CIRS Dynamic Thorax phantom (CIRS Inc. Norfolk, VA) programmed with nominal motion patterns. A 16 cm foam cube was mounted perpendicular to the system camera axis on the phantom's motion actuator (Figure 1a). The camera's principal axis was aligned parallel to the couch surface using its integrated inertial measurement unit (IMU), aligning it with the DICOM Z‐axis (IEC 61217; patient axis). Longitudinal displacements were measured using the camera's depth sensor data, while vertical and lateral displacements were measured using optical data (lateral data was not considered but are acquired in a similar manner to vertical displacements). Single‐dimension movements (longitudinal or vertical) were actuated with known sinusoidal motions of various amplitudes (5–30 mm) and frequencies (6, 12, 15, and 20 cycles/min) at depths ranging from 560 to 1120 mm to evaluate system performance against the programmed motion. The proposed system's measurement latency was tested against a synchronized Sick laser displacement sensor with accuracy: ≤0.45 mm ± 0.4 mm, acquisition rate:1 kHz (Sick Inc. Waldkirch, Germany). Latency was defined based on phase shift calculation as previously demonstrated.^43^

### 2.4 Motion tracking of 3D irregular surface

To simulate a real‐patient setup, we affixed a triangle sticker on a mannequin within the camera's field of view. Securing the mannequin's inferior edge to the table and allowing the CIRS motion phantom actuator to slide along its posterior aspect created complex motion mimicking respiratory movements in both anterior/posterior (AP) and superior/inferior (SI) directions simultaneously (Figure 1b). The programmed motion was a reproduction of a volunteer respiratory trace recording including two deep inspiration breath‐holds alternating with periods of regular breathing over 110 s. Lacking ground truth for complex motion data, we benchmarked our system against the Varian IDENTIFY™ (version 2.6, Varian Medical Systems, Palo Alto, CA) optical imaging system operating at 3–10fps. Simultaneous data acquisition with both systems captured the 3D irregular breathing simulated on the irregular rigid surface.

### 2.5 Statistical analysis

The offline analysis validated the proposed system against ground truth using tracking success rate (TSR) and mean absolute error (MAE) metrics.^47^ TSR measured the percentage of frames where motion deviated less than 2, 1.5, and 1 mm from nominal data, while MAE evaluated deviations between recorded and nominal motion at paired measurement points. Pearson correlation coefficients (*r*) were calculated for each 1D‐motion tracking session to assess the linear relationship between recorded and input motion. For the same sessions, Bland–Altman analysis examined consistency between the camera‐based system and the established commercial optical imaging system.^48^, ^49^ To compare the two systems, camera data was interpolated to match the timestamps of the IDENTIFY™ data, ensuring paired measurements. Because vendor limitations to data access, the proposed system could not be interfaced with the clinical computers. Therefore, small phase shift corrections were applied where interpolation artifacts induced any systematic time offset between. Bland–Altman analysis identified systematic bias by evaluating motion differences relative to the average values from both clinical reference and the camera‐based measurements.

## 3 RESULTS

The camera's static depth accuracy was evaluated at various distances. At 560 mm, it demonstrated a MAE of 0.03 mm with a SD of ± 0.13 mm; at 1120 mm, the MAE increased to 0.65 mm with an SD of ± 0.61 mm over 1‐min acquisitions. These results compare favorably with a prior study on the D415 model, which reported standard deviations ranging from <0.2 mm at 400 mm to ∼2.5–3 mm at 1200 mm,^43^ suggesting that our implementation using the D455 sensor achieves improved precision at clinically relevant distances. Minimal drift was observed during prolonged measurements at 560 mm, with a standard deviation of 0.52 mm (0.35 mm for the first 10 min), indicating consistent performance.

**FIGURE 2:**
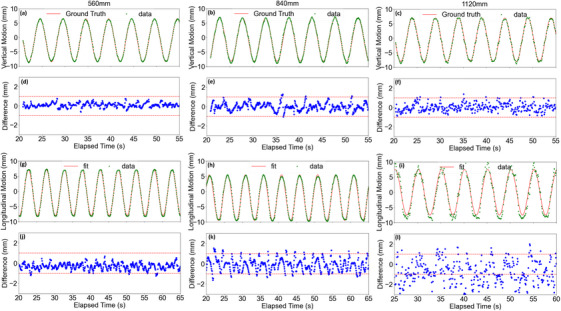
Measurement of vertical and longitudinal 1D motion of a flat surface at predefined distances from the camera. Panels (a), (b), and (c) show vertical displacements, and panels (g), (h), and (i) show longitudinal displacements at distances of 560, 840, and 1120 mm, respectively, at a frequency of 0.20 Hz. Panels (d), (e), and (f) display the difference between measured and ground truth vertical motion, while panels (j), (k), and (l) show the difference between measured and fitted longitudinal motion.

The system effectively tracked vertical and longitudinal 1D motions of a flat surface at distances of 560, 840, and 1120 mm, as illustrated in Figure 2. At 560 mm, the vertical tracking closely matched the ground truth (panel a), with minimal deviations and a tracking success rate (TSR) of 100% for deviations ≤1 mm (panel d). Although increased distance led to slightly higher noise levels (panels e and f), the system maintained strong performance, demonstrating reliable tracking at 840 and 1120 mm. At 560 mm, longitudinal tracking achieved a TSR of 99.4% for deviations ≤2 mm and 92% for deviations ≤1 mm (panel j). As the distance increased, a gradual rise in error magnitude was observed (panels k and l), consistent with the expected decline in accuracy.

**FIGURE 3:**
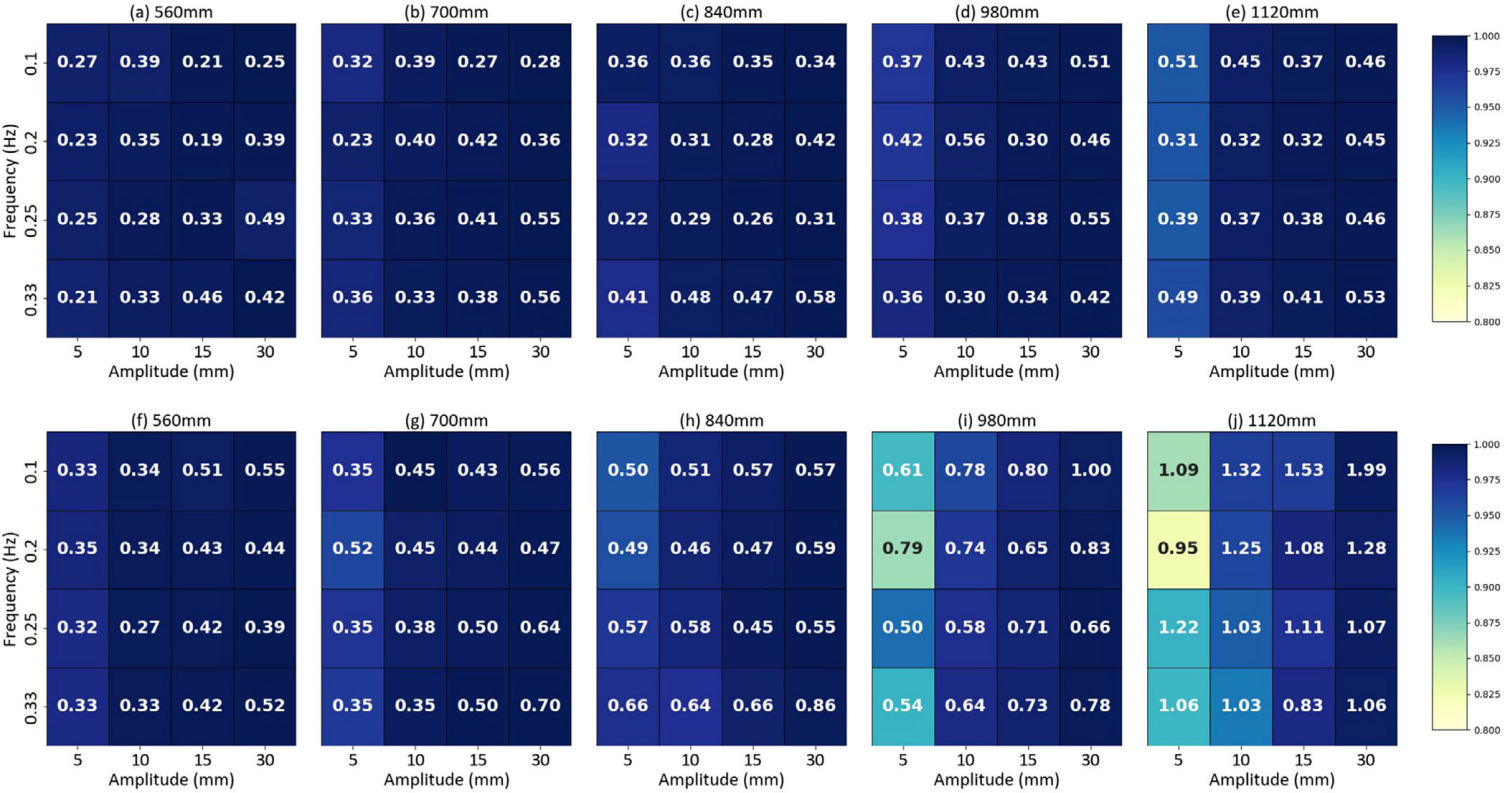
Pearson correlation heatmaps and mean absolute error (MAE) values for vertical and longitudinal motion tracking. Panels (a) through (e) depict vertical motion tracking, and panels (f) through (j) show longitudinal motion tracking at various distances.

Heatmaps of Pearson correlation coefficients and MAE revealed high correlation values (> 0.99) at shorter distances and larger amplitudes, as shown in Figure 3a–e for vertical motion and Figure 3f–j for longitudinal motion. Correlation decreased with increasing distance; at 1120 mm, vertical tracking correlations ranged from 0.95 to 0.99, while longitudinal tracking ranged from 0.82 to 0.99 for most scenarios. The system tracked moving targets above 10 Hz with a latency of 60.73 ms (± 0.74 ms), verified using a 10 mm amplitude sine wave at 0.25 Hz.

**FIGURE 4:**
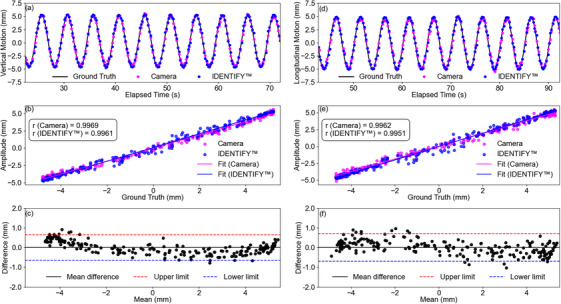
Comparison of vertical and longitudinal motion tracking data for a flat surface. Panels (a) and (d) show motion over time, comparing camera and IDENTIFY™ system data with the ground truth. Panels (b) and (e) present Pearson correlation analyses between the ground truth and both systems, including correlation coefficients (r) and linear fits. Panels (c) and (f) display Bland–Altman plots illustrating agreement between the camera and IDENTIFY™ systems.

**FIGURE 5:**
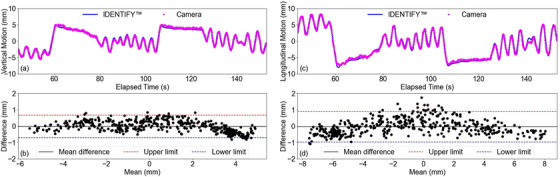
Comparison of motion tracking performance between the IDENTIFY™ system and the camera system at 560 mm. Panels (a) and (c) display vertical and longitudinal motion data over time, respectively. Panels (b) and (d) show Bland–Altman plots for vertical and longitudinal motions, highlighting the mean differences and limits of agreement.

Comparisons with the IDENTIFY™ system showed both systems closely followed the ground truth signal (programmed motion), as illustrated in Figure 4. Pearson correlation coefficients were high for both vertical and longitudinal motions (*r* ≈ 0.99). Bland–Altman analysis indicated negligible systematic bias, with mean differences near zero and uncertainties less than 1 mm. In multi‐dimensional motion tests simulating complex respiratory movements, both systems demonstrated high accuracy as shown in Figure 5.

Table 1 provides a detailed breakdown of the Bland–Altman analysis, showing mean differences close to zero, with limits of agreement increasing with depth but remaining within acceptable ranges. For vertical motion, limits increased from ± 0.67 mm at 560 mm to ± 1.29 mm at 1120 mm; for longitudinal motion, limits increased from ± 0.91to ± 4.13 mm over the same depth range.

**TABLE 1:** Bland–Altman analysis comparing the camera system data with a commercial optical surface imaging system for 3D motion of an irregular, non‐flat surface. Depth indicates the distance between the camera and the surrogate marker affixed to the irregular surface.

	Vertical motion		Longitudinal motion	
Depth (mm)	Bias	Limits	Bias	Limits
560	−0.01 ± 0.35 mm	0.67 mm, −0.7 mm	−0.03 ± 0.48 mm	0.91mm, −0.98 mm
700	0.02 ± 0.32 mm	0.66 mm, −0.61 mm	−0.05 ± 0.55 mm	1.04 mm, −1.13 mm
840	−0.02 ± 0.52 mm	1.0 mm, −1.05 mm	−0.03 ± 1.00 mm	1.98 mm, −2.03 mm
980	0.00 ± 0.60 mm	1.19 mm, −1.18 mm	0.06 ± 1.42 mm	2.83 mm, −2.72 mm
1120	−0.03 ± 0.68 mm	1.29 mm, −1.35 mm	0.00 ± 2.11 mm	4.13 mm, −4.13 mm

## 4 DISCUSSION

We proposed a realtime optical surface imaging respiratory motion tracking system combining affordable depth camera technology with custom software. Validated using dynamic phantoms with various breathing‐like motions including simple periodic motion and recorded irregular signals from a healthy volunteer. The system captured data at amplitudes and frequencies representative of typical human breathing.

Unlike the LiDAR approach,^36^, ^37^, ^38^ which can only capture vertical motion, flat surface experiments confirmed the system's ability to track absolute motion in three dimensions. Lateral motion was not analyzed in the full scope of the testing framework since lateral displacements are not typically tracked in respiratory motion monitoring using external surrogates.^2^ By aligning the camera axis to the couch longitudinal axis allowed for acquiring motion data in absolute displacements within DICOM coordinates, thus being able to reference the programmed 1D motion as the ground truth.

For patient or healthy volunteers tracking scenarios, where ground truth cannot be established, we benchmarked our system against the clinical IDENTIFY™ system by concomitantly tracking a single target. Bland–Altman analysis demonstrated an expectation value for agreement within ± 1.5 mm, capturing 95% of paired measurements for vertical motion at 560 mm, close to the IDENTIFY™ isocenter. Additionally, by removing the need for specific smartphone models and custom mounts,^38^ it offers a more practical and scalable solution, particularly for under‐resourced radiation oncology departments where accessibility and accuracy are critical.

Compared to previous studies using depth cameras for radiotherapy motion monitoring.^39^, ^43^ Our system maintains higher accuracy across similar distances, errors of 0.03 ± 0.13 mm at 560 mm and 0.65 ± 0.61 mm at 1120 mm while offering realtime capabilities without extensive offline processing. Unlike prior systems requiring multiple data refinement steps,^43^ our framework facilitates testing for ensuing clinical applications. Another study using the Breathe Well (Respiratory Innovations Pty Ltd, Sydney, Australia) system showed that accuracy decreases on curved surfaces and with irregular breathing, which could limit its effectiveness in highly dynamic clinical environments.^39^ In contrast, our system demonstrated good accuracy on curved surfaces and during irregular breathing, particularly in the vertical direction, where limits of agreement remained within acceptable ranges.

The system meets AAPM TG‐302 minimum requirements for geometric accuracy,^27^ achieving a mean absolute error (MAE) under 2 mm in all cases and under 1 mm for distances between 560 and 980 mm, where it also meets the more stringent ESTRO‐ACROP requirements, ensuring high accuracy in motion tracking.^50^ Vertical motion showed smaller variability compared to longitudinal motion. Although accuracy decreases at distances greater than 1 m, consistent with previous reports.^43^ The vertical component remains accurately tracked, while longitudinal accuracy diminishes. In the clinical implementation of the IDENTIFY^TM^ system, the breath holds monitoring function only tracks vertical surface displacement, thus highlighting that this dimension is most sensitive in the considered clinical scenario. Since this is designed to be a couch mounted system, positioning the camera between 400 and 800 mm is feasible without interfering with clinical workflows. Although total drift is within AAPM TG‐302 and ESTRO‐ACROP recommendations, increased distances may elevate errors; however, keeping acquisition times short mitigates temperature‐induced drift. The system is designed to process image pre‐processing and Mask R‐CNN detection and segmentation in real‐time. It operates efficiently with low latency and can accommodate additional processing, such as interfacing with peripherals for beam‐hold decisions, without exceeding recommended limits.^2^, ^27^ Additionally, the system meets the ESTRO‐ACROP latency requirement of ≤200 ms.^50^

Commercial systems such as AlignRT, Catalyst, and IDENTIFY™ rely on three ceiling‐mounted stereovision pods, proprietary projectors, and vendor‐locked workstations; consequently, they sell at six‐figure list prices and require permanent room infrastructure. By contrast, our prototype is built from a single Intel RealSense D455 depth camera (≈ US $250) and an off‐the‐shelf GPU, keeping the total hardware outlay below US $5000. The camera bolts to a couch‐indexed arm that can be installed or removed in minutes and replicated across treatment rooms without additional licenses, making the platform inherently scalable for resource‐limited centers. It is worth mentioning here that commercial systems also provide similar performance characteristics but are set for much larger focal distances vs. our prototype, which as mentioned needs to maintain a smaller distance to the tracked surface. The camera is also much smaller and simpler in design in our prototype when compared to the commercial implementation.

Technically, the frugal design does not compromise performance. Across 560–980 mm stand‐offs the system maintains sub‐millimeter mean error and <2 mm at 1.12 m while delivering an end‐to‐end latency of 61 ± 1 ms, thereby satisfying the 2 mm (general) and 1 mm (SRS/SBRT) accuracy thresholds and 200 ms latency guidance set by AAPM TG‐302. Bland–Altman limits of agreement indicate that the tracked motion is similar to that measured with Varian Identify under identical phantom motion, suggesting the low‐cost architecture matches commercial precision at clinically relevant distances. To support clinical translation, an invention disclosure has been filed with the Office of Technology Transfer, and a spin‐out partnership is under exploration to commercialize the couch‐mounted hardware and developed software for underserved clinics.

These findings underscore the potential of our system as a cost‐effective yet high‐performing alternative to established optical surface imaging solutions. Advanced commercial optical surface imaging systems are available,^25^ but their high costs limit adoption, especially in regions with financial constraints.^35^ Our cost‐effective depth camera‐based prototype addresses these disparities, offering a promising alternative for areas with limited resources. Despite the high accuracy, real‐time capabilities and low cost of our system, there are some limitations to consider. One significant limitation is the decrease in motion tracking accuracy with increased distance. Although the system maintains submillimeter accuracy at closer distances, the error margin increases notably as the distance from the camera increases. Specifically, longitudinal motion tracking shows more pronounced errors compared to vertical motion due to increased noise at longer distances. This noise can be attributed to the limitations in depth resolution and the inherent challenges in capturing precise longitudinal movements. Although matrix transformations can mitigate some noise effects in the vertical direction, the longitudinal noise remains a challenge.^43^

Another limitation is related to the tracking ROI. If the tracked object exits the ROI, the system suspends tracking and prompts the user to reset the tracking area. This mechanism is designed to avoid false positives and drift, but it also requires the object to remain consistently within the defined ROI posing a constraint during larger respiratory excursions or patient movement. It must also be noted that, as currently designed, this system does not encompass the full suite of set‐up and workflow integration features provided by clinical SGRT systems. Commercial SGRT platforms are deeply integrated into treatment room environments and support a wide range of clinical workflows. Although such functionality is a goal for future development, it is not currently implemented in our system.

The original camera stand is not optimized for clinical settings; therefore, a versatile, modular, couch‐mounted camera stand is being designed to enhance positional flexibility and minimize gantry interference. This new design will maintain the camera within the target zone of 560–780 mm, allowing for effective depth resolution without obstructing gantry rotation or therapist workflow. Initial testing has shown no gantry interference, and upcoming healthy volunteer trials will include a dry run to confirm clearance and compatibility. Future work will build on the extensive phantom‐based experiments, which helped finalize our framework and demonstrate safety prior to engaging human volunteers. This strategy not only minimized the time required from participants but also facilitated IRB approval by showing a safe, well‐refined methodology. We are now poised to launch a study with 20 healthy volunteers having secured ethical clearance and a custom‐designed, couch‐mounted camera stand that preserves the camera's optimal line of sight while adapting to individual setups. This phase will be followed by prospective patient data collection to further validate the system in clinical settings. In parallel, we aim to expand upon the current Mask R‐CNN architecture, training it to recognize anatomical landmarks for precise 3D positioning and real‐time feedback. By integrating additional machine learning algorithms capable of generating detailed 3D point clouds, we seek to offer comprehensive respiratory‐motion visualization and a clear path toward clinical certification. Such refinements emphasize the system's real‐world applicability and affordability, ultimately broadening access to accurate, patient‐centric respiratory monitoring in radiotherapy.

## 5 CONCLUSION

Our study demonstrates the potential of a low‐cost, depth camera‐based respiratory tracking system integrated with Mask R‐CNN for precise respiratory motion management in radiotherapy. The system consistently achieved high accuracy, providing surface tracking accuracy of less than 2 mm at shorter distances, as required by AAPM TG‐302. By addressing financial and technological barriers, this prototype aims to make advanced respiratory motion management more accessible, particularly in regions with limited resources. As part of the next phase, we plan to evaluate the system's performance in a prospective study involving healthy volunteers, enabling assessment of inter‐subject variability and usability in more realistic clinical scenarios. Additional work will focus on refining the camera stand design for broader treatment compatibility, enhancing patient positioning support for real‐time radiotherapy and improved patient outcomes.

## AUTHOR CONTRIBUTIONS

Mandeep Kaur was responsible for the software development, experimental setup, data collection, and manuscript writing. Marian Axente conceived the project idea, helped with the development and experimental setup, and contributed significantly to the manuscript review and editing. Hania Al‐Hallaq reviewed the manuscript and provided valuable feedback.

## CONFLICT OF INTEREST STATEMENT

The authors declare no conflicts of interest.
